# Detection and phylogenetic characterization of arbovirus dual-infections among persons during a chikungunya fever outbreak, Haiti 2014

**DOI:** 10.1371/journal.pntd.0006505

**Published:** 2018-05-31

**Authors:** Sarah K. White, Carla Mavian, Maha A. Elbadry, Valery Madsen Beau De Rochars, Taylor Paisie, Taina Telisma, Marco Salemi, John A. Lednicky, J. Glenn Morris

**Affiliations:** 1 Emerging Pathogens Institute, University of Florida, Gainesville, Florida, United States of America; 2 Department of Environmental and Global Health, College of Public Health and Health Professions, University of Florida, Gainesville, Florida, United States of America; 3 Department of Pathology, College of Medicine, University of Florida, Gainesville, Florida, United States of America; 4 Department of Health Services Research, Management and Policy, College of Public Health and Health Professions, University of Florida, Gainesville, Florida, United States of America; 5 Christianville Foundation School Clinic, Gressier, Haiti; 6 Department of Medicine, College of Medicine, University of Florida, Gainesville, Florida, United States of America; Institute for Disease Modeling, UNITED STATES

## Abstract

In the context of recent arbovirus epidemics, questions about the frequency of simultaneous infection of patients with different arbovirus species have been raised. In 2014, a major *Chikungunya virus* (CHIKV) epidemic impacted the Caribbean and South America. As part of ongoing screening of schoolchildren presenting with acute undifferentiated febrile illness in rural Haiti, we used RT-PCR to identify CHIKV infections in 82 of 100 children with this diagnosis during May—August 2014. Among these, eight were infected with a second arbovirus: six with *Zika virus* (ZIKV), one with *Dengue virus* serotype 2, and one with *Mayaro virus* (MAYV). These dual infections were only detected following culture of the specimen, suggesting low viral loads of the co-infecting species. Phylogenetic analyses indicated that the ZIKV and MAYV strains differ from those detected later in 2014 and 2015, respectively. Moreover, CHIKV and ZIKV strains from co-infected patients clustered monophyletically in their respective phylogeny, and clock calibration traced back the common ancestor of each clade to an overlapping timeframe of introduction of these arboviruses onto the island.

## Author summary

*Chikungunya virus* (CHIKV) was the cause of a major epidemic in Haiti from May through August, 2014, as the disease swept through the Caribbean and South and Central America. During the time-period of the epidemic, our group was monitoring a cohort of children in semi-rural Haiti, using molecular and virology methods to assess the etiology of acute undifferentiated febrile illness. We identified CHIKV infections in 82 of 100 children presenting with fever during this time-period; six of the children infected with CHIKV were found to be co-infected with *Zika virus* (ZIKV*)*, one with *Dengue virus* serotype 2, and one with *Mayaro virus*. For the CHIKV and ZIKV infections, a molecular clock calibrated framework revealed results suggestive of simultaneous co-infection. Our data highlight the frequency of dual infections in arbovirus disease cases (with the possibility of simultaneous co-infection), and document the frequent movement of virus strains within the Caribbean and the American continents.

## Introduction

*Chikungunya virus* (CHIKV) (family *Togaviridae*, genus *Alphavirus*), is the causative agent of chikungunya fever. After first isolation of CHIKV in 1952 in present-day Tanzania, outbreaks and epidemics were limited to regions of Asia, Africa, and the Pacific Islands. In 2013, CHIKV emerged for the first time in the Americas, with sustained autochthonous transmission and rapid spread through the region [1–3]. The acute symptoms of CHIKV infection are similar to those of infection with other arbovirus species, including *Dengue virus* (DENV), *Zika virus* (ZIKV), and *Mayaro virus* (MAYV), each presenting with a constellation of symptoms including fever, headache, and myalgias/arthralgias. Long-term, CHIKV infections have been linked with persistent arthralgias in a subset of cases[3]; it has also been reported that upwards of 90% of CHIKV-infected individuals are symptomatic, in contrast to findings with ZIKV, where it is estimated that only 20% of infected persons are symptomatic [4,5].

The similarity of the clinical presentation of acute-phase arbovirus infections is further complicated by the potential for simultaneous infection with multiple arboviruses. In a recent literature review, co-infections with CHIKV and DENV ranged from 1% to 34% of patients [6]. However, virtually no data are available on frequency of co-infection with CHIKV and arboviruses other than DENV. Even where good laboratory diagnostic facilities are available, identification of co-infections often does not occur, as there is a tendency to cease investigation once an initial pathogen has been identified, and/or identification of a second pathogen may require facilities for virus isolation, which may not be available.

As part of ongoing studies of acute undifferentiated febrile illness in a cohort of school children in rural Haiti, we identified 82 children with RT-PCR-confirmed CHIKV infections during May-August 2014, corresponding with the time period when the Caribbean CHIKV epidemic was moving through Haiti. Specimens were also simultaneously screened by RT-PCR for DENV1-4, then additionally for ZIKV. Aliquots of the plasma specimens were then inoculated onto cell cultures for the isolation of additional pathogens of potential interest [6]. We report here results of these studies, focusing on rates of arbovirus co-infection in our patient cohort and potential sources of origin of the co-infecting strains.

## Methods

### Patient population/specimen collection

Blood specimens were collected from schoolchildren with acute febrile illness seen at the Christianville School clinic in Gressier, Haiti, beginning in May 2014. This clinic serves as the primary source of healthcare for approximately 1,250 children at four school campuses (the main LaSalle campus [campus A] and three small satellite elementary school campuses [campuses B, C, and D]) operated by the Christianville Foundation in the Gressier/Leogane region; schools are located within a radius of approximately 10 miles. The clinic has facilities for short-term stays of a few hours for sick children, with those requiring hospitalization referred to the local community hospital. As previously described, acute undifferentiated febrile illness was defined as occurrence of fever in a child with no obvious source of infection (i.e., no respiratory symptoms, symptoms of UTI, or evidence of malaria or typhoid) [7]. We have previously reported isolation of ZIKV [8], DENV [9], *Human coronavirus* NL63 [10], and *Enterovirus* D68 [11] from children in this school cohort; however, the cases/outbreaks in these prior publications did not include CHIKV, or cases within the May-August, 2014, time frame of the current study. Clinical features of these cases are reported elsewhere [12].

Whole blood (ca. 0.5–2 ml) was collected in K_2_EDTA tubes (BD Vacutainer, Becton, Dickinson and Company, Franklin Lakes, NJ). As part of the initial diagnostic evaluation, plasma was screened for CHIKV viral RNA (vRNA) by molecular methods [13,14]. All virus isolations and RNA extractions on CHIKV-positive specimens were performed in a BSL-3 facility at the University of Florida (UF) Emerging Pathogens Institute in Gainesville, Florida.

### Ethics statement

The UF IRB and the Haitian National IRB approved all protocols, and written informed consent was obtained from parents or guardians of all study participants.

### Isolation of CHIKV in cell cultures

To assess the sensitivity of RT-PCR tests and for confirmation purposes, CHIKV isolations from plasma specimens were attempted in epithelioid cells derived from African Green Monkey kidneys (Vero E6, ATCC CRL-1586). The Vero E6 cells used for virus isolation were maintained in cell culture medium comprised of aDMEM (advanced Dulbecco’s modified essential medium) supplemented with 10% low antibody, heat-inactivated, gamma-irradiated fetal bovine serum (FBS, Hyclone, GE Healthcare Life Sciences, Pittsburgh, PA), L-alanine and L-glutamine supplement (GlutaMAX, Invitrogen, Carlsbad, CA), and 50 μg/ml penicillin, 50μg/ml streptomycin, 100μg/ml neomycin (PSN antibiotics, Invitrogen) with incubation at 37°C in 5% CO_2_. Confluent cell cultures were split into 25cm^2^ flasks 24 hours prior to inoculation to attain 60% confluent cell monolayers the following day. Prior to inoculation, the culture medium was removed and inoculum containing 100μl of plasma that had been filtered through a sterile 0.45 filter and mixed with 400μL aDMEM with 3% FBS, GlutaMAX, and PSN antibiotics was added to the monolayer. The inoculated monolayer was incubated at 37°C in 5% CO_2,_ and rocked every 15 minutes for 1 hour. A negative control (mock-infected) cell culture was inoculated with 500ul of DMEM without virus or plasma and handled in parallel with the other cultures. After allowing for virus adsorption for 1 hr, the inocula were removed and replaced with 3ml of aDMEM with 3% FBS, GlutaMAX, and PSN antibiotics, and thereafter incubated at 37°C in 5% CO_2_. Cell cultures were refed every 3 days by the replacement of 1.5ml of spent media with aDMEM with 3% FBS. The cultures were observed for up to 21 days’ post-inoculation, or until CHIKV-induced cytopathic effects (CPE) were observed in the cell monolayers using an inverted microscope. When CPE were observed throughout 50% of the monolayer, a final collection of 2ml spent media, and 1ml of scraped cells in spent media, were cryopreserved at -80°C for future tests.

### Molecular detection

Total RNA was extracted from both plasma specimens and spent media, and tested by real-time RT-PCR (rtRT-PCR) following published protocols for CHIKV vRNA [13] for confirmation of previous tests performed in Haiti. RNA extracted from plasma specimens were then screened for DENV serotypes 1–4 [15] and ZIKV [16] vRNAS by rtRT-PCR. Cycle threshold (Ct)-values under 38 were considered positive. Viral genomic RNA that was extracted from CHIKV, DENV1-4, and ZIKV strains that were obtained from the Biodefense and Emerging Infections Research Resource Repository (BEI Resources, Manassas, VA) were used as positive control materials for rtRT-PCR. Cell cultures were also tested for the presence of DENV and ZIKV vRNAs by rtRT-PCR, even if the corresponding plasma specimen tested negative. Additionally, spent media from cultures displaying non-CHIKV CPE, that were DENV and ZIKV negative by rtRT-PCR, were screened with a duplex RT-PCR for the vRNAs of other alphaviruses (*Venezuelan equine encephalitis -*, *Eastern equine encephalitis -*, *Western equine encephalitis -*, *Aura—*and *Mayaro viruses*) and flaviviruses (*Yellow fever -*, *Saint Louis encephalitis -*, *Bussaquara -*, *Ilheus -*, *and Rocio viruses*) [17]. The DENV-1 strain from BEI and the 2015 MAYV sample from our laboratory [18] were used as the flavivirus and alphavirus positive controls, respectively, in the duplex RT-PCR protocol.

### Sequencing of virus genomes

Whole genome sequence data from 10 of the CHIKV samples (7 from children with co-infections, 3 from selected randomly mono-infections) were obtained by Sanger sequencing and a primer-walking approach, as previously described [14,19]. Similarly, we designed sequencing primers for MAYV and ZIKV that also amplify approximately 800bp overlapping segments, and used a primer walking method for whole genome sequencing of those viruses [8,18]. For the sequencing of DENV, primers described by Christenbury *et al* were utilized [20]. Amplification of each segment was performed using an Accuscript high-fidelity first-strand cDNA synthesis kit (Agilent Technologies, Santa Clara, CA) followed by PCR with Phusion polymerase (New England Biolabs, Ipswich, MA). The 5’ and 3’ ends of the viral genomes were obtained using RNA-ligase mediated (RLM) systems for 5’ and 3’ Rapid Amplification of cDNA Ends (RACE) per the manufacturer’s protocols (Life Technologies, Carlsbad, CA). Amplicons were purified, sequenced bi-directionally, then the sequences assembled with the use of Sequencher DNA sequence analysis software v2.1 (Gene Codes, Ann Arbor, MI), and subsequently analyzed in comparison to DENV, MAYV, and ZIKV sequences available in GenBank for nucleotide sequence comparisons. The vRNA sequences we obtained differed from those of the corresponding viruses in our repository, confirming the newly analyzed sequences did not arise from laboratory contamination.

### Full genome sequence data sets and maximum likelihood phylogenetic inference

Alignments for each virus-specific dataset (CHIKV, DENV-2, MAYV, and ZIKV), including full genome sequences selected to represent the major clades described so far for each virus were obtained using the MUSCLE algorithm implemented in MEGA7 (http://www.megasoftware.net/) [21–23]. GenBank accession numbers (AC), geographical location, and year associated with isolation of each strain are reported in S1 Table. Based on previous evidence of recombination reported for MAYV, potential presence of recombination was also investigated for the new 2014 MAYV isolate (KY985361) [24] with algorithms implemented in the RDP4 [25] software (http://web.cbio.uct.ac.za/~darren/rdp.html), as previously described [24]. The phylogenetic signal in each virus-specific data set was evaluated by likelihood mapping using TreePuzzle (http://www.tree-puzzle.de/)[26], and substitution saturation plots using DAMBE6 ((http://dambe.bio.uottawa.ca/DAMBE/dambe.aspx) [27].

Maximum likelihood (ML) trees were inferred by IQ-TREE using the best-fitting nucleotide substitution model chosen according to Bayesian Information Criterion (BIC) (S2 Fig) [28,29]. Statistical robustness for internal branching order in each phylogeny was assessed by UFBoot—Ultrafast Bootstrap (BB) Approximation (2,000 replicates) implemented in IQ-TREE [30], and strong statistical support along the branches was defined as BB>90%. UFBoot was eployed as it accelerates computing and reduces overestimating branch support [30]. Alignments files are available from the authors upon request.

### Bayesian inference of time-scaled phylogenies

The temporal signal for each dataset was assessed using ML trees with TempEst v1.5 (http://tree.bio.ed.ac.uk/software/tempest/) [31]. Bayesian inference of time-scaled phylogenies were carried out with BEAST v1.8.4 (http://beast.bio.ed.ac.uk/) [32,33] by enforcing either a strict or relaxed molecular clock [34] and the SDR06 substitution model with empirical base frequencies and gamma distribution of site-specific rate heterogeneity. Two demographic priors were also tested for each analysis: constant population size or non-parametric Bayesian Skyline Plot (BSP). Bayesian Markov Chain Monte Carlo (MCMC) were run for 50–200 million generations (sampled at fixed intervals to obtain posterior distributions with 10,000 data points), depending on the data set, in order to assure proper mixing of the MCMC, which was assessed on the basis of the effective sampling size (ESS) of each parameter estimate, accepting only ESS values >200. The best clock/demographic model for each virus-specific data set was chosen by comparing marginal likelihood estimates (MLE) [35], obtained using path sampling (PS) and stepping-stone sampling (SS) methods [33,36] with Bayes Factor (BF) (S2 Table). In practice, the BF natural logarithm was used for comparison with *ln*BF<2 indicating no evidence against the null hypothesis (i.e. less parameter-rich model), 2–6—weak evidence, 6–10—strong evidence, and >10 very strong evidence [37].

For each data set, the maximum clade credibility (MCC) tree (tree with the largest product of posterior clade probabilities) was selected from the posterior tree distribution of the best fitting clock/demographic model, after appropriate burn-in, using TreeAnnotator v1.8.4 implemented in the BEAST software package. The final trees were manipulated in FigTree v1.4.3 for display purposes (http://tree.bio.ed.ac.uk/software/figtree/). XML files for the BEAST runs are available from the authors upon request.

## Results

With one exception, all rtRT-PCR-confirmed CHIKV infections in our student cohort occurred in the 9-week period between May 23 and July 25, 2014; the one “outlier” case occurred in mid-August, 2014. Among the 100 plasma specimens taken from febrile schoolchildren in the May-August time period, 82 tested positive via rtRT-PCR for CHIKV, but were negative for DENV1-4 and ZIKV. Among the 18 specimens from febrile schoolchildren during this time period that were not positive for CHIKV, two gave equivocal results in the rtRT-PCR assay and were excluded from the analysis, two were DENV type 1 positive, and 14 were negative in all assays. Cultures were not performed on these 18 specimens due to insufficient specimen volume, reflecting the large number of assays that had been conducted in the initial screening of what were relatively small blood samples from each child. The mean age of children from whom specimens were collected was 9.6 years. Ninety-two of the 100 specimens were from students at Campus A of the school; 7 were from Campus B, and one was from Campus C. Gender, average age, and school level for the 82 children whose samples were positive for CHIKV by rtRT-PCR are included in Table 1.

**Table 1:** Number of children from whom specimens were obtained which were positive for CHIKV, by gender, average age, and school level.

*variable*	*Number (n = 82)*
*GENDER*	
male	44
female	38
*AVERAGE AGE*	9.4 years
*SCHOOL LEVEL*	
Kindergarten	21
Primary school	41
Secondary school	20

### Virus isolation

**Fig 1:**
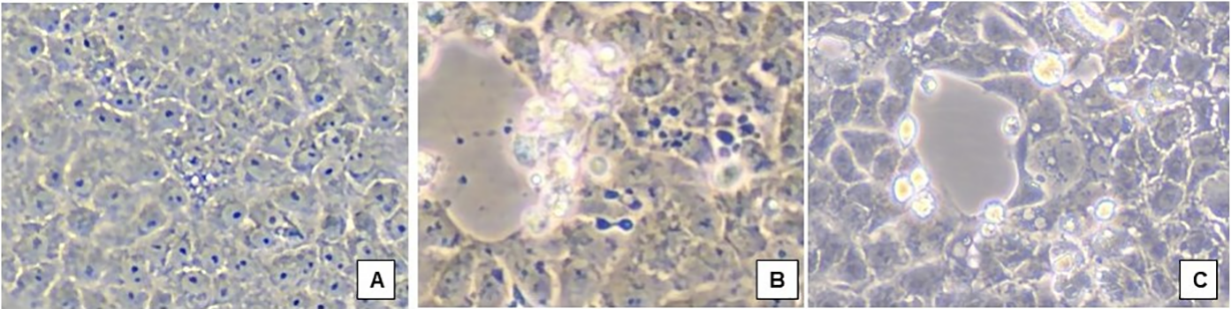
Chikungunya virus-induced CPE in Vero E6 cells with filtered plasma specimens. (A) Mock-infected Vero E6 cells, and (B) and (C), CHIKV-induced CPE in Vero E6 cells inoculated with CHIKV-positive plasma samples. All images at 200X original magnification.

**Fig 2:**
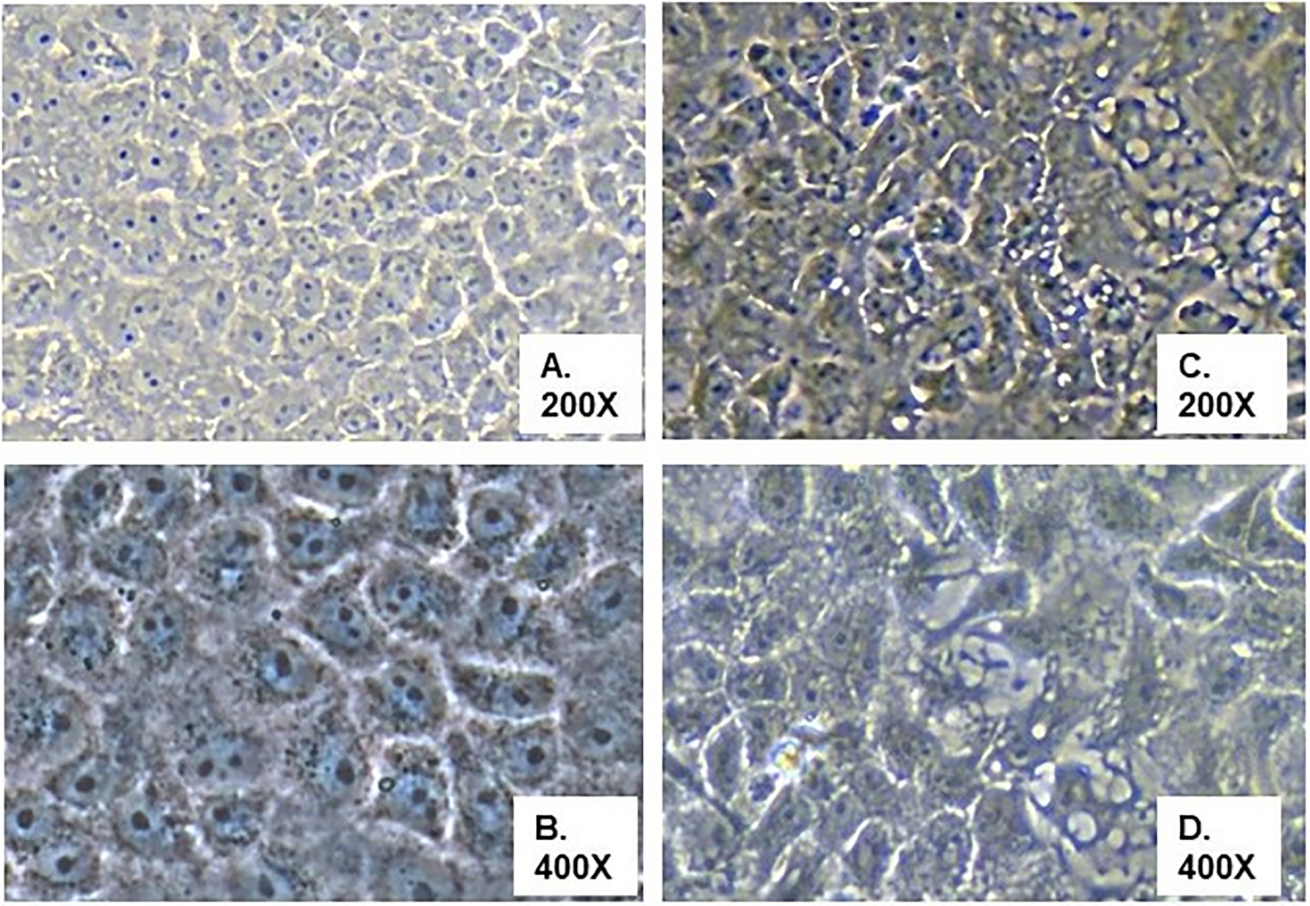
Mixed CPE in Vero E6 cells inoculated with filtered plasma specimens. Panels (A) and (B), mock-infected Vero E6 cells. Panels (C) and (D), Vero E6 cells inoculated with virus-containing plasma. Original image magnifications are indicated beneath the panel identification letters.

Of the 82 rtRT-PCR CHIKV-positive specimens, attempts were made to isolate virus from 62 specimens, with the remainder not inoculated onto cell cultures due to insufficient specimen volume. Typical CHIKV-induced CPE, consisting of cell membrane blebbing, cell lysis, and apoptosis, were observed in cultures from 43 of the 62 samples on average 5 days post inoculation (dpi), with some specimens displaying advanced CPE as early as 2 dpi and others not until 20 dpi (Fig 1); target vRNA’s were positive by rtRT-PCR for CHIKV but negative for DENV and ZIKV [15–17]. All negative control cell cultures lacked CPE and tested negative by rtRT-PCR for the target vRNAs of this work. Eleven (15%) of the 62 samples cultured did not display any CPE during the period of culture and tested negative for target vRNAs in these studies, despite initial positive CHIKV results by rtRT-PCR. Eight cultures displayed CPE inconsistent with those expected for CHIKV (Fig 2). The mixed CPE in these latter cultures was associated with co-infection with CHIKV together with another virus, as determined by molecular tests of the cultured specimens: co-infecting arboviruses included ZIKV (n = 6), DENV type 2 (n = 1), and MAYV virus (n = 1). Sex and age of patients were comparable for those with CHIKV mono-inections and the subset of CHIKV cases with co-infections. Seven of the 8 co-infections were from Campus A of the school; one co-infection (CHIKV and ZIKV) was in a student from Campus B.

### Phylogenetic and molecular clock analyses

**Fig 3:**
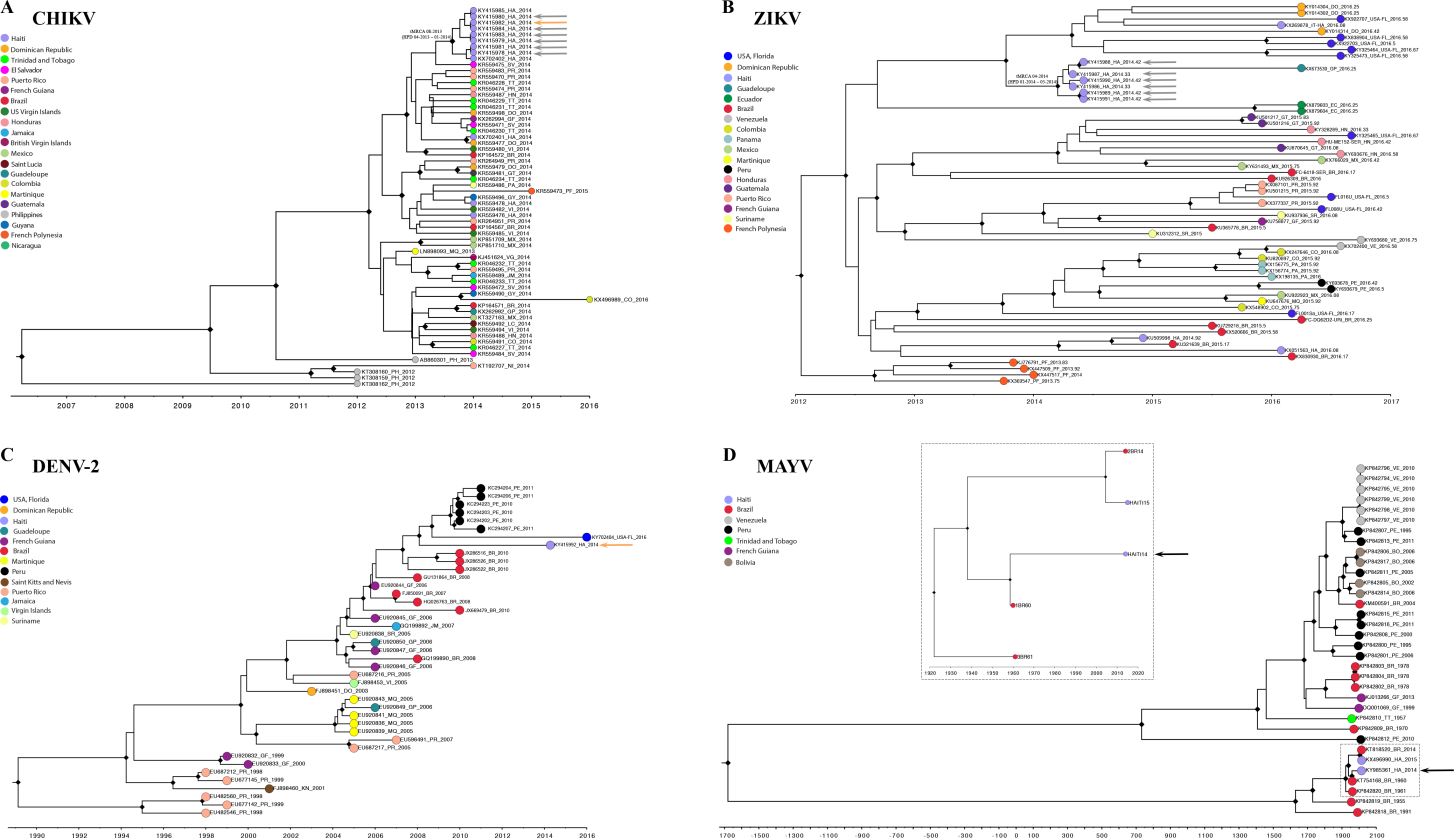
Time-scaled Bayesian phylogenetic maximum clade credibility (MCC) trees of arbovirus strains. MCC trees for (A) CHIKV, (B) ZIKV, (C) DENV-2 and (D) MAYV were inferred from full genome sequences using BEAST v1.8.4. Gray arrows indicate strains from CHIKV and ZIKV co-infected patients, orange from CHIKV and DENV-2, black from CHIKV and MAYV (sequence for CHIKV strain from the co-infected patient was not determined). Letters next to arrows indicate ID of co-infected patients. Tips are colored by sampling location as indicated in the legends to the left of each tree; diamonds at each node indicate strong statistical support along branches defined as posterior probability > 0.9. For MAYV (D), insert box shows relationship of recent Haitian and Brazilian strains.

CHIKV, ZIKV, MAYV and DENV-2 alignments displayed strong phylogenetic signal (S1 Fig), as well as temporal signal sufficient for the calibration of an accurate molecular clock (S2 Fig). ML trees with branch lengths scaled in nucleotide substitutions per site (S3 Fig), as well as time-scaled Bayesian phylogenies (Fig 3) using the best fitting molecular clock/demographic model (S2 Table), were inferred for each data set. Bayesian and ML reconstructions displayed very similar topologies and suggested three independent phylogenetic lineages of CHIKV in Haiti, possibly the result of three separate introductions (Figs 3A and S3A). The CHIKV strains from the current study were in a well-supported monophyletic clade which was most closely related to a strain from El Salvador (Fig 3A). Within this clade, six CHIKV strains were from patients co-infected with ZIKV and one with DENV-2. Molecular clock median estimate of the time of the most recent common ancestor (tMRCA) for the clade was August 2013, with 95% highest posterior density (95%HPD) intervals between April 2013 and January 2014 (Fig 3A).

As shown in Fig 3, two other 2014 CHIKV isolates, not collected as part of this study but previously reported by Lanciotti (KR559476 and KR559478) from mono-infected Haitian patients, clustered within a second distinct clade related to different Central and South American strains, but separate from our Gressier strains. One of the 10 sequenced CHIKV strains from our cohort, from a randomly selected mono-infection in a child from Campus B, was in yet a third clade, which clustered most closely with a strain from the Dominican Republic.

The six ZIKV sequences obtained in June 2014 from the CHIKV co-infected patients were highly similar (99.9%) to each other and also cluster within a well-supported monophyletic clade, which, interestingly, includes a divergent strain isolated in 2016 from Guadaloupe (Figs 3B and S3B). The primary clade of co-infecting ZIKV strains is closely related to another ZIKV clade including isolates from the 2016 USA Florida outbreak [38]. In contrast, previously reported [8] Haitian strains from this same school cohort in December of 2014 (KU509998), and Haitian strains from early 2016 (KX051563 [39] and KX269878 [40]) belonged to distinct phylogenetic lineages, consistent with multiple independent introductions of ZIKV to Haiti. The median origin of the Haitian ZIKV co-infection strains was estimated by molecular clock in April 2014 with a 95%HPD between January and May 2014 (Fig 3B), a timeframe overlapping with the estimates of the corresponding CHIKV co-infections clade.

The specimen from the CHIKV and DENV-2 co-infection was obtained on June 11, 2014. The DENV-2 isolate was closely related to a previously reported strain isolated in early 2016 (KX702404) from a US traveler returning from Haiti [39] and they both cluster within a well-supported clade including Brazilian and Peruvian subclades (Figs 3C and S3C). According to the time-scaled phylogeny (Fig 3C), the tMRCA of the DENV-2 isolate from the CHIKV co-infected patient traced back to February 2008 with 95%HPD between May 2007 and October 2008.

Finally, the MAYV strain from a CHIKV co-infected patient seen on June 4, 2014 is the earliest documented case of MAYV in Haiti to date. Its genome is highly similar (99.4%) to a strain from Brazil isolated in 1960, and phylogenetic analysis cluster both strains in a well-supported monophyletic clade (Figs 3D and S3D), within genotype L [24,40], with a tMRCA dating back to 1958 (95%HPD interval of 1949–1960). It should be noted, however, that since we only have one MAYV strain clustering with the Brazilian 1960 strain, the tMRCA is unlikely to represent the date of introduction of MAYV in Haiti. Another, previously reported Haitian strain (KX496990) [18,24], clusters in a different well-supported clade within genotype L, a scenario once again consistent with multiple independent introductions of the virus in the Caribbean.

## Discussion

Despite increasing recognition of the frequency with which simultaneous co-infection with multiple arbovirus species occurs [19,39,41–43], co-infection dynamics are not well understood, and the clinical, pathologic, and immunologic significance of such co-infections remains to be determined. As a starting point in unraveling these dynamics, we were interested in better quantifying the frequency of such infections, and exploring possible origins of co-infecting strains. To optimize our ability to identify co-infections (and following standard practices in our laboratory [8,17,19,39]), we continued diagnostic studies even after initial identification of CHIKV or another pathogen in a patient sample. Interestingly, while we identified multiple co-infecting viruses in cell culture, the original patient rtRT-PCR in this study was, in each instance, negative for the second pathogen, suggesting that viral loads for the co-infecting species were low. We also had instances where viral cultures were negative in the setting of a positive rtRT-PCR, possibly reflecting the presence of non-viable virus in the sample, and/or strains that have mutated or which do not grow well in Vero cells. If we are to better understand the role of co-infections in disease occurrence, the diagnostic significance of both rtRT-PCR negative/culture positive and rtRT-PCR positive/culture negative samples will require further study.

Co-infections with CHIKV and DENV are well described, with reported rates of co-infection ranging from 1% to 34% [6]; the highest reported co-infection rates were from Madagascar (26%) and Nigeria (34%). Another study from the Colombian-Venezuelan border reports a CHIKV/DENV co-infection prevalence of 7.6% [43]. In our study, in contrast, the co-infection rate was only 1%, with only two additional dengue cases (both DENV-1) identified among CHIKV-negative patients; this may reflect a low level of circulating DENV in the community during the CHIKV epidemic, and/or a high existing level of population immunity to the virus. We also identified one CHIKV/MAYV co-infection. We have previously reported a mixed MAYV and DENV-1 infection that occurred outside of the time frame of the CHIKV epidemic (January, 2015) in the same school cohort [18]. The high genomic similarity of this Haitian MAYV isolate with a Brazilian strain isolated in 1960 corroborates our hypothesis that MAYV has been criptically circulating in the human population at a sub-epidemic level, most likely in Brazil, for years and that it was introduced just recently onto Hispaniola [24].

What was unexpected in our data set was the relatively high frequency of ZIKV/CHIKV co-infections. This occurred in a setting in which we were only screening plasma samples. As we have previously reported [39], urine can be positive for ZIKV for a longer period of time than plasma; if we had also screened urine, there is the possibility that we would have identified additional ZIKV-positive patients. We, and others, have previously reported cases of ZIKV/CHIKV co-infection [19,42,44,45], so finding the two together is not surprising. The ZIKV case cluster in the current study would appear to have been relatively widespread within the community (i.e., not a point source at the school), as one of the six co-infections was from a child at a different school campus, ca. 5 miles from the main campus. Interestingly, our molecular clock analysis indicates that the strains of CHIKV and ZIKV found in our patients were both introduced in Haiti within the same short time frame, each one giving rise to a well-supported monophyletic clade, one including all CHIKVs from CHIKV/ZIKV co-infected subjects, the other one including all ZIKVs from the same co-infected subjects. The simultaneous emergence of these two clades in the Haitian population is compatible with a simultaneous co-infection scenario or indicate, at the very least, the sequential infection of a patient with two arboviruses within a relatively short period of time. In light of recent reports that mosquito co-infection with ZIKV and CHIKV allows simultaneous transmission without affecting vector competence [46], it is plausible that the two viruses were co-transmitted by the same mosquito. Given that these are among the earliest, if not the earliest, documented ZIKV infections in the hemisphere, the observation that they appeared together with epidemic CHIKV raises interesting questions about the possible role of CHIKV in the initiation of the ZIKV epidemic in the Americas.

Because our work draws on student populations from four schools located within a radius of about 10 miles, we have some feel for disease prevalence within the immediate study area; however, generalizability of these data to larger regions of Haiti may be limited. Nonetheless, our findings are consistent with the idea that there were multiple introductions of CHIKV into Haiti between 2013 and 2014, with identification of three distinct phylogenetic lineages, each clustering with strains from different parts of the Caribbean and/or South America. This was also the case for ZIKV: the ZIKV co-infecting strains did not cluster with the ZIKV strains previously obtained in Haiti in 2014 and 2016, consistent with multiple introductions or re-introductions of this arbovirus in Haiti between 2014 and 2016. Although for DENV-2 and MAYV the data are more limited, the tMRCAs for DENV-2 suggested that the introduction in Haiti happened between 2008 and 2014, a time interval again overlapping with the estimated introduction of both CHIKV and ZIKV co-infecting strains circulating in Haiti. Clustering of both the ZIKV and DENV-2 strains together with ZIKV and DENV-2 strains obtained in Florida in 2016 [38,47], as well as the multiple independent introductions of CHIKV, ZIKV, and MAYV to Haiti inferred from the phylogenies, demonstrate the potential role of the Caribbean as a node for arbovirus infections connecting South, Central, and North America.

## Supporting information

S1 Checklist STROBE checklist. (DOCX)

S1 Fig Substitution saturation and likelihood mapping analysis of CHIKV, ZIKV, DENV-2, and MAYV full genome sequence data sets. (A) Scatter plots of pairwise nucleotide transition (s) and transversion (v) substitutions versus genetic distance [48] and (B) likelihood mappings showing supports for each of three alternative topologies (corners of the triangle) of 10,000 randomly selected groups of four sequences (quartets), unresolved quartets (center of the triangle), and partly resolved quartets (edges of the triangle) for CHIKV, ZIKV, DENV-2, and MAYV sequences.
(PDF)

S2 Fig Analysis of temporal signal in CHIKV, ZIKV, DENV-2, and MAYV full genome sequence datasets. Plots represent root-to-tip genetic distance in the ML phylogeny versus sampling time for each taxa, (A) CHIKV, (B) ZIKV, (C) DENV-2 and (D) MAYV. The correlation coefficient “r” is reported for each linear regression.
(PDF)

S3 Fig Maximum likelihood (ML) selected arbovirus phylogenies inferred from full genome sequence alignments obtained using the IQ-TREE software. Midpoint rooted ML phylogenies for (A) CHIKV, (B) ZIKV, (C) DENV-2 and (D) MAYV. Branch lengths are scaled in nucleotide substitutions per site according to the bar at the bottom of each tree. Tips are colored by sampling location as indicated in the legends to the left of each tree; diamonds at each node indicate strong statistical support along branches defined as ultrafast bootstrapping>90% (out of 2,000 replicates).
(PDF)

S1 Table *Zika virus*, *Dengue virus type 2*, and *Mayaro virus* genomic sequence information. (DOCX)

S2 Table Bayes factor (BF) comparison of nested molecular clock and Bayesian demographic models. (DOCX)
