# Supplementary material for: Detection and phylogenetic characterization of arbovirus dual-infections among persons during a chikungunya fever outbreak, Haiti 2014
Source: PLoS Negl Trop Dis. 2018 May 31;12(5):e0006505. doi: 10.1371/journal.pntd.0006505 (PMC5997359; doi:10.1371/journal.pntd.0006505)
Supplement: S1 Checklist — (DOCX) [file pntd.0006505.s001.docx]

S1 Checklist. STROBE Statement

Item No Recommendation

1. Title and abstract (a) Clinical cohort

(b) Abstract lines26-29

**Introduction**

2. Background/rationale Introduction, lines 53-75

3. Objectives Introduction, lines 77-79

**Methods**

4. Study design Methods, lines 81-93

5. Setting Methods, lines 81-93

6. Participants (a) Methods, lines 82-89

(b) Not a matched study

7. Variables Methods lines 82-98

8. Data sources/measurement Methods, lines 82-142

9. Bias Only presenting virology results in the present report, methods lines 144-160

10. Study size Methods, lines 83-86

11. Quantitative variables Not applicable for presented data

(a) Not applicable for presented data

(b) Not applicable for presented data

(c) Results, lines 211-213

(d) Not applicable

12. Statistical methods (e) No statistics on the presented findings

**Results**

13. Participants (a) Results, lines 204-213

(b) Results, lines 211-213

(c) Flow diagram not applicable

14. Descriptive Data (a) Results, lines 213-215

(b) Results, lines 211-213

(c) Follow-up time not applicable

15. Outcome data Results, lines 204-2013

16. Main results

(a) Results, lines 213-214

(b) No continuous variables were categorized

(c) Not relevant, to translate estimates of relative risk into absolute risk

17. Other analyses Results, lines 216-281

**Discussion**

18. Key results Discussion, lines 302-308

19. Limitations Discussion, lines 288-295

20. Interpretation Discussion, lines 283-342

21. Generalisability Discussion, lines 317-342

**Other information**

22. Funding NIAID R01 AI26357-01S1 to JGM, and pilot grant from the University of Florida
