# Supplementary figures and images for: Detection and phylogenetic characterization of arbovirus dual-infections among persons during a chikungunya fever outbreak, Haiti 2014
Source: PLoS Negl Trop Dis. 2018 May 31;12(5):e0006505. doi: 10.1371/journal.pntd.0006505 (PMC5997359; doi:10.1371/journal.pntd.0006505)

A

CHIKV

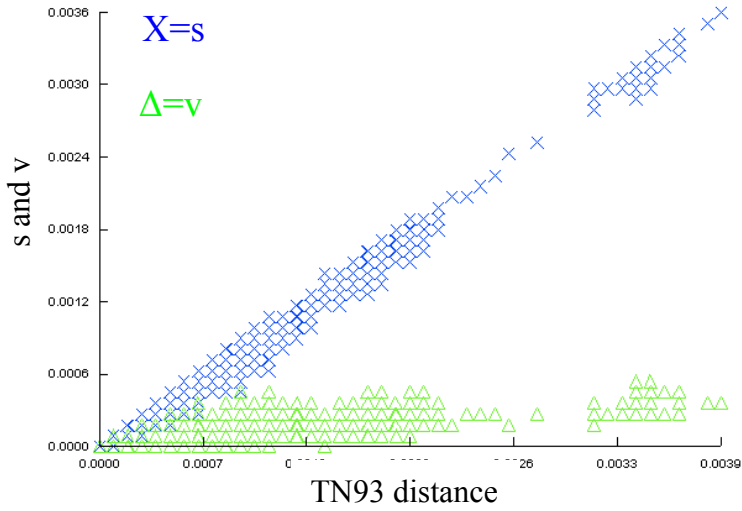

ZIKV

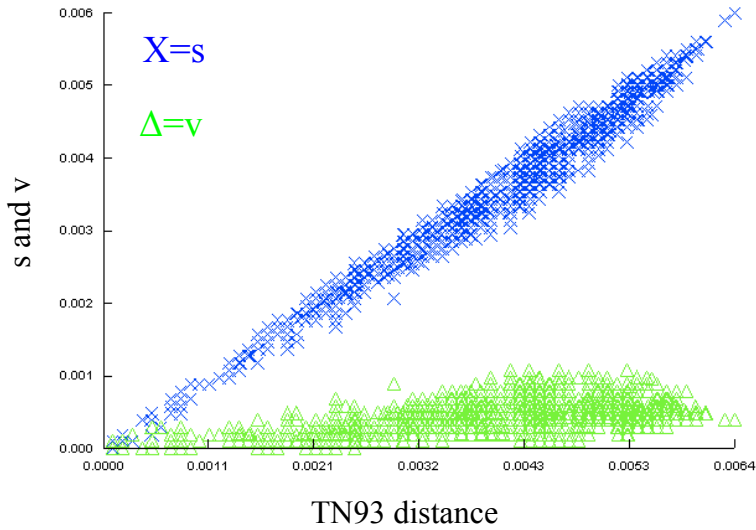

DENV-2

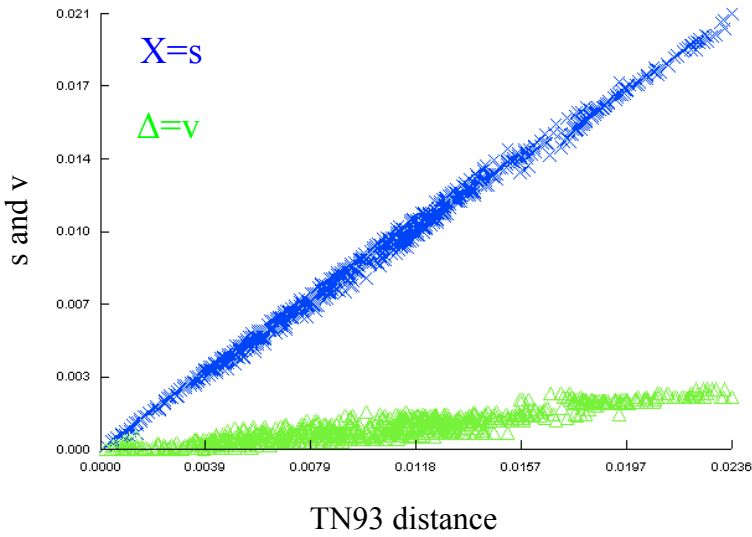

MAYV

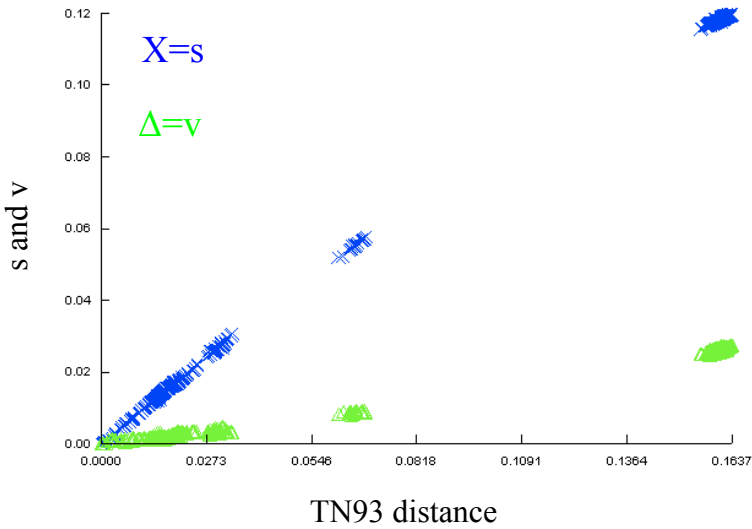

B

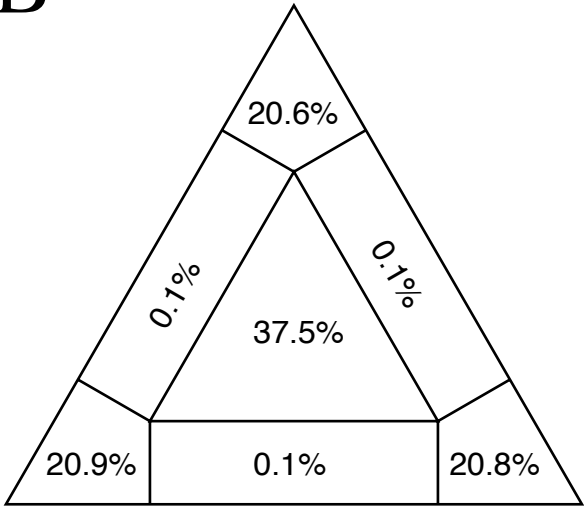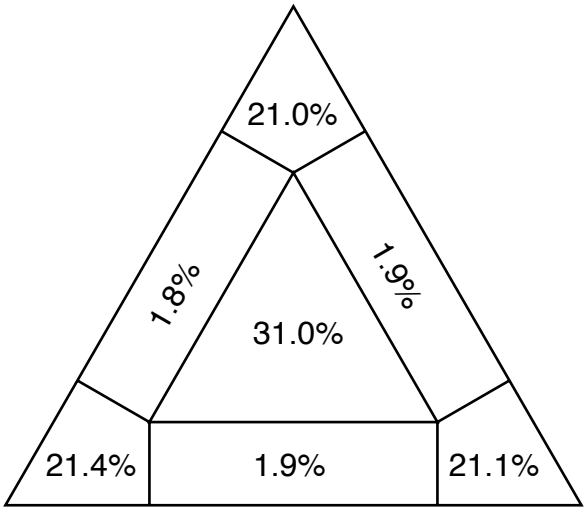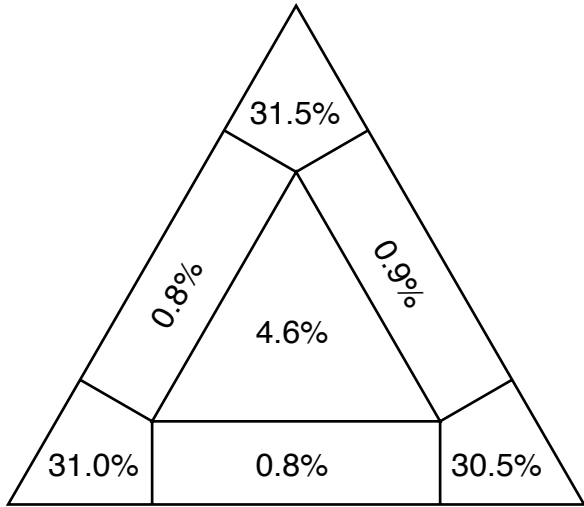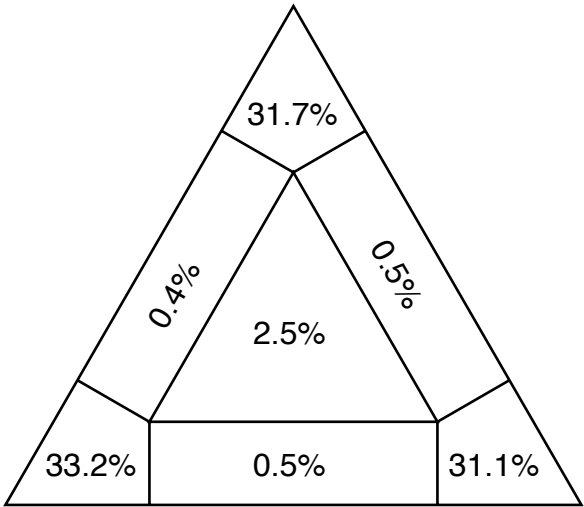

Supplement: S1 Fig — (A) Scatter plots of pairwise nucleotide transition (s) and transversion (v) substitutions versus genetic distance [48] and (B) likelihood mappings showing supports for each of three alternative topologies (corners of the triangle) of 10,000 randomly selected groups of four sequences (quartets), unresolved quartets (center of the triangle), and partly resolved quartets (edges of the triangle) for CHIKV, ZIKV, DENV-2, and MAYV sequences. (PDF) [file pntd.0006505.s002.pdf]

A CHIKV

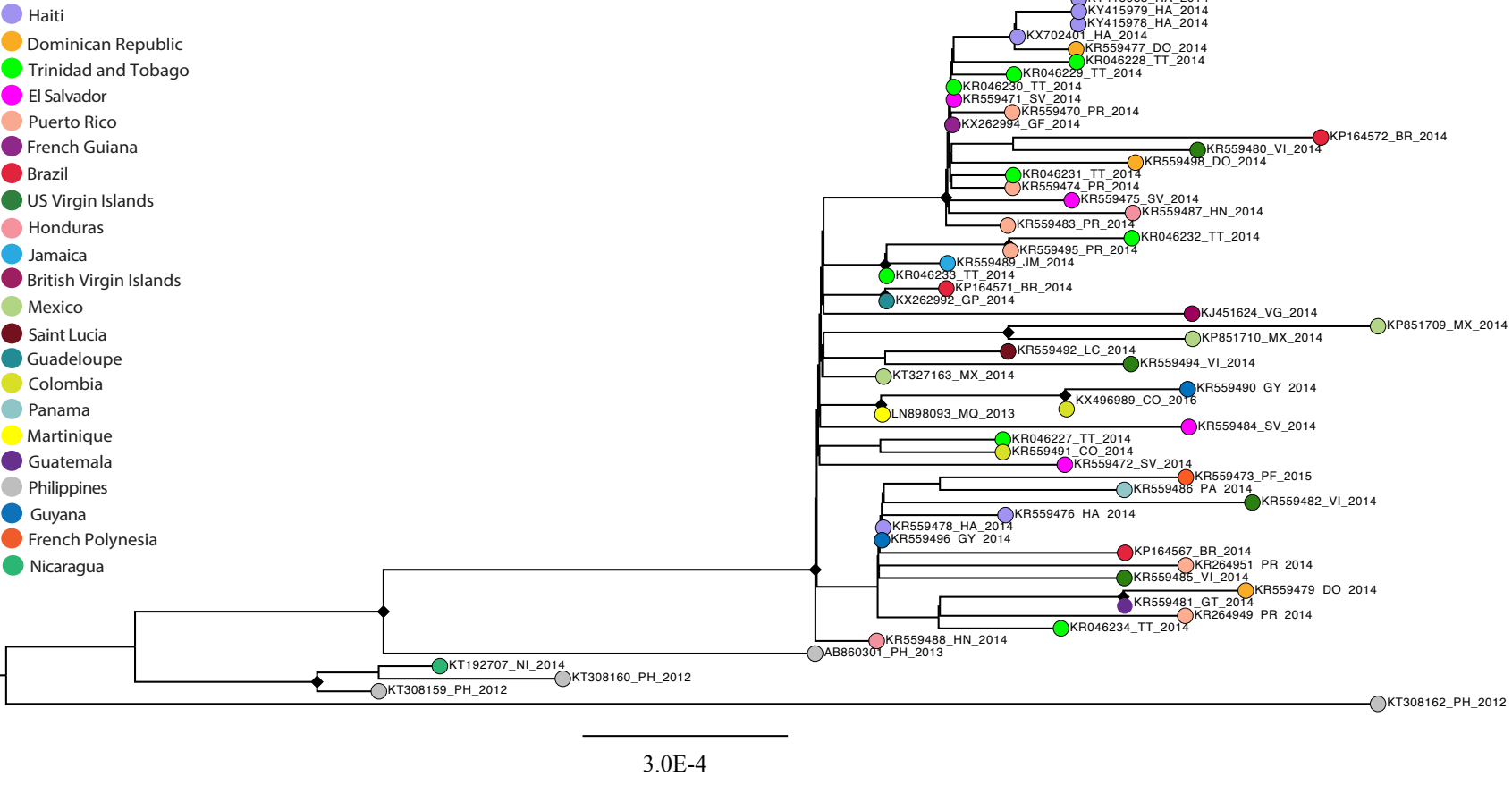

C DENV-2

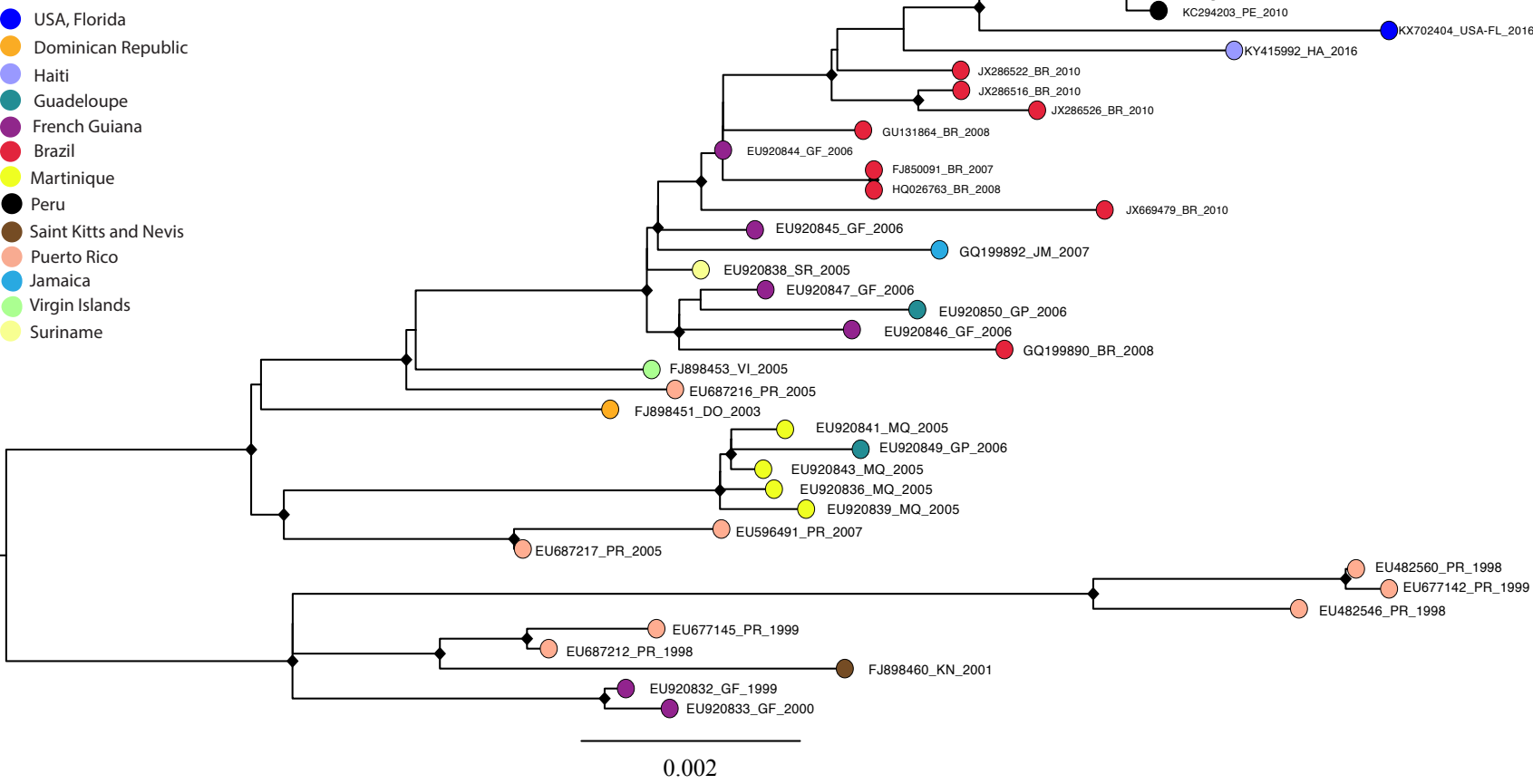

B ZIKV

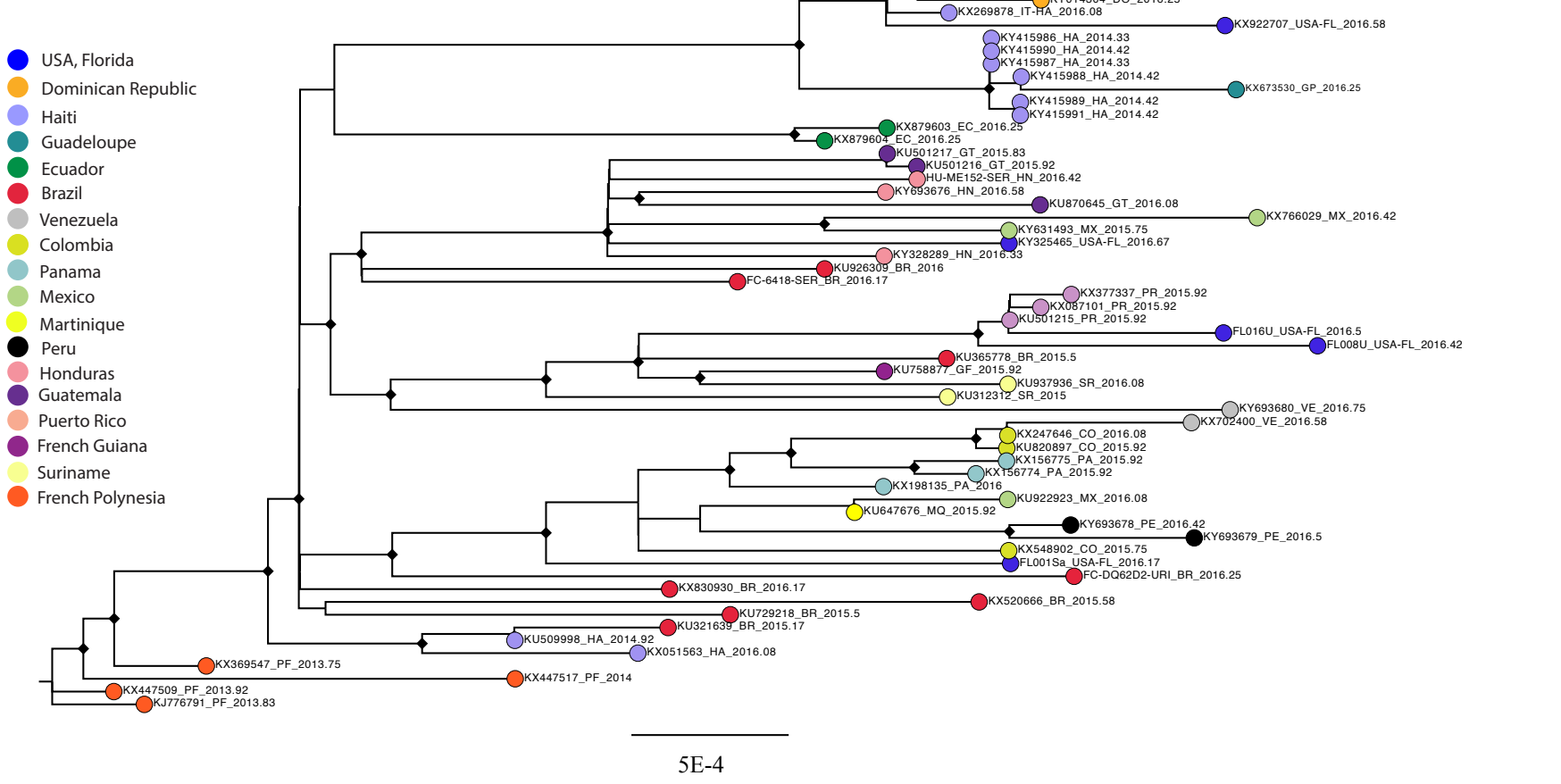

D MAYV

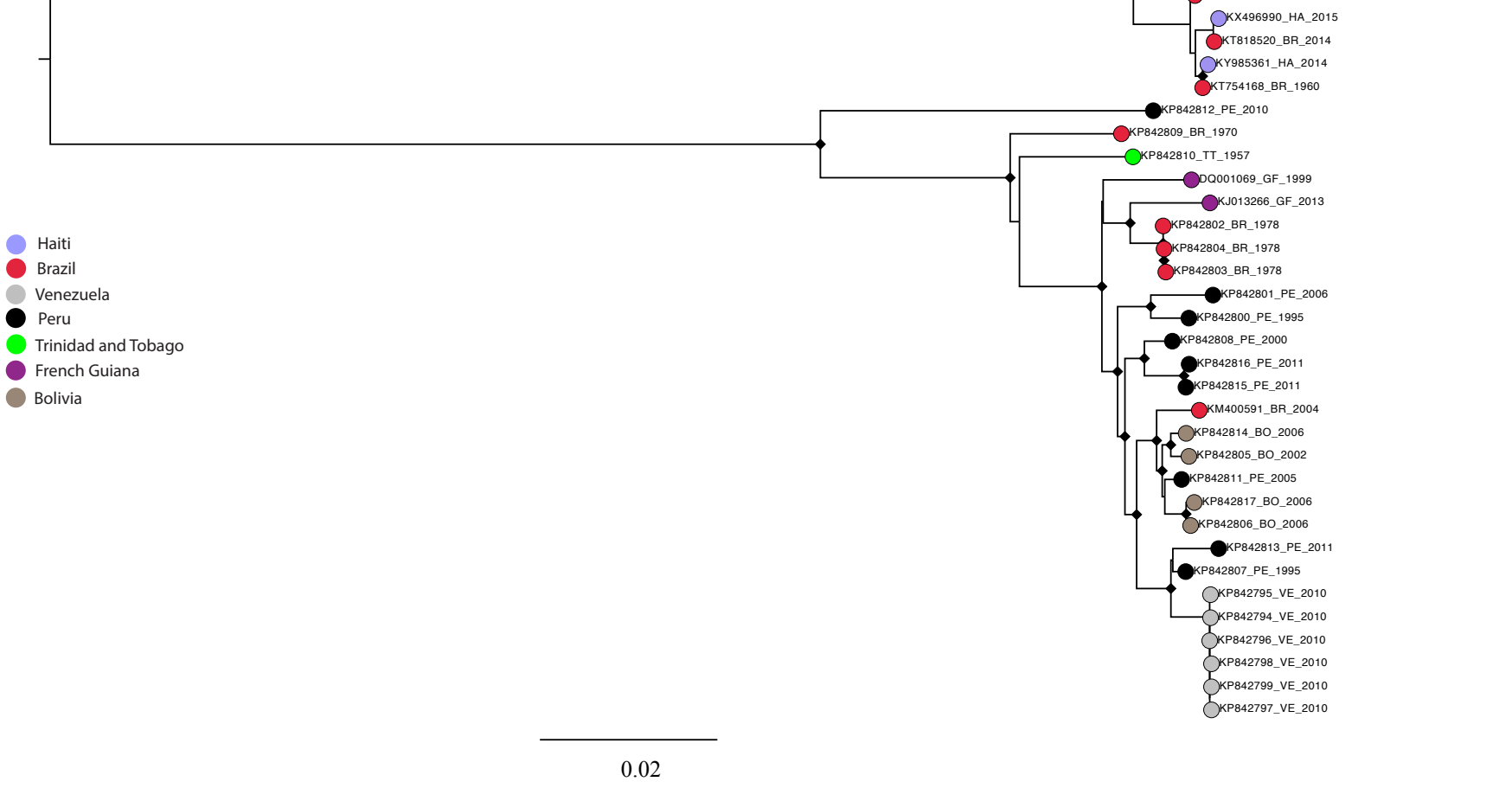

Supplement: S2 Fig — Plots represent root-to-tip genetic distance in the ML phylogeny versus sampling time for each taxa, (A) CHIKV, (B) ZIKV, (C) DENV-2 and (D) MAYV. The correlation coefficient “r” is reported for each linear regression. (PDF) [file pntd.0006505.s003.pdf]

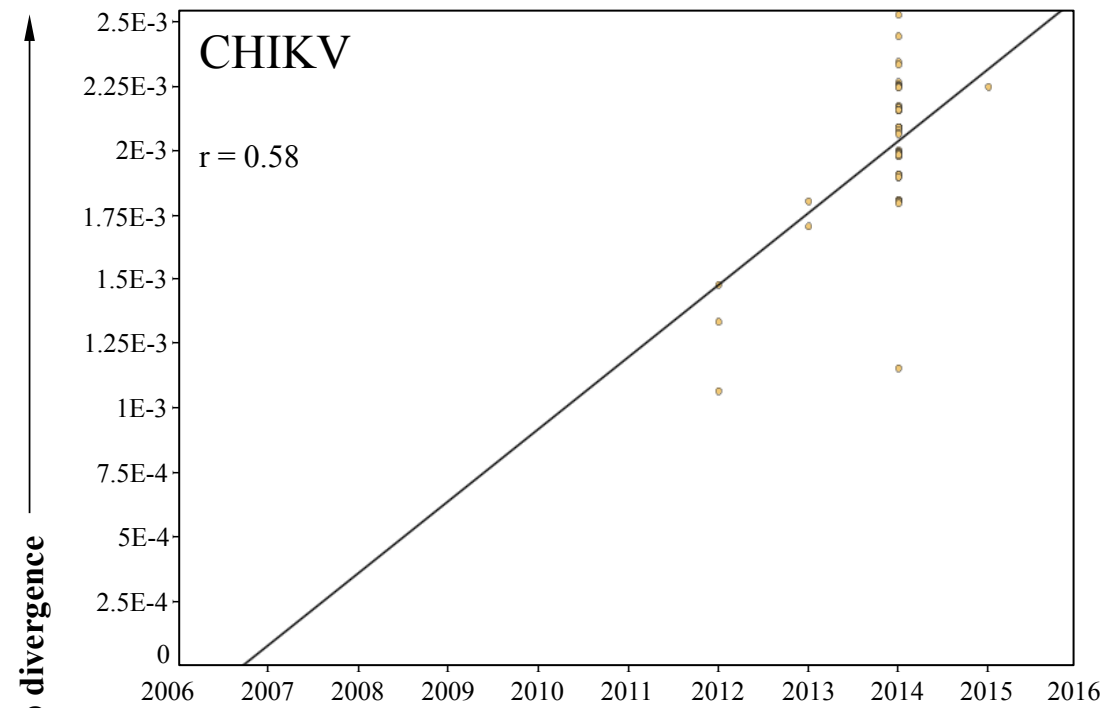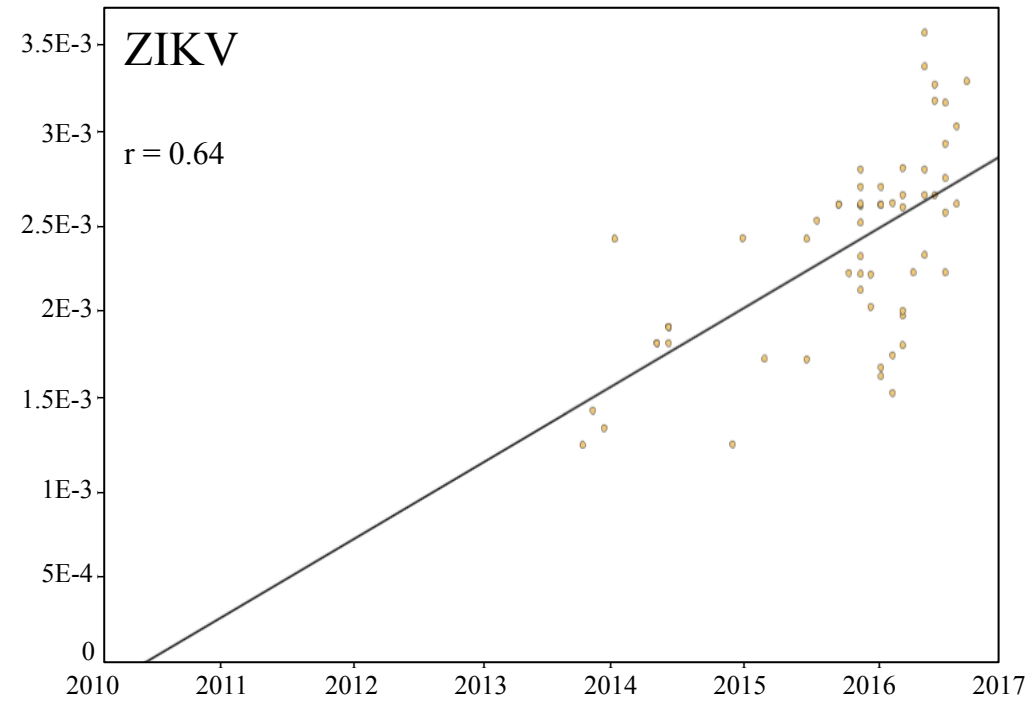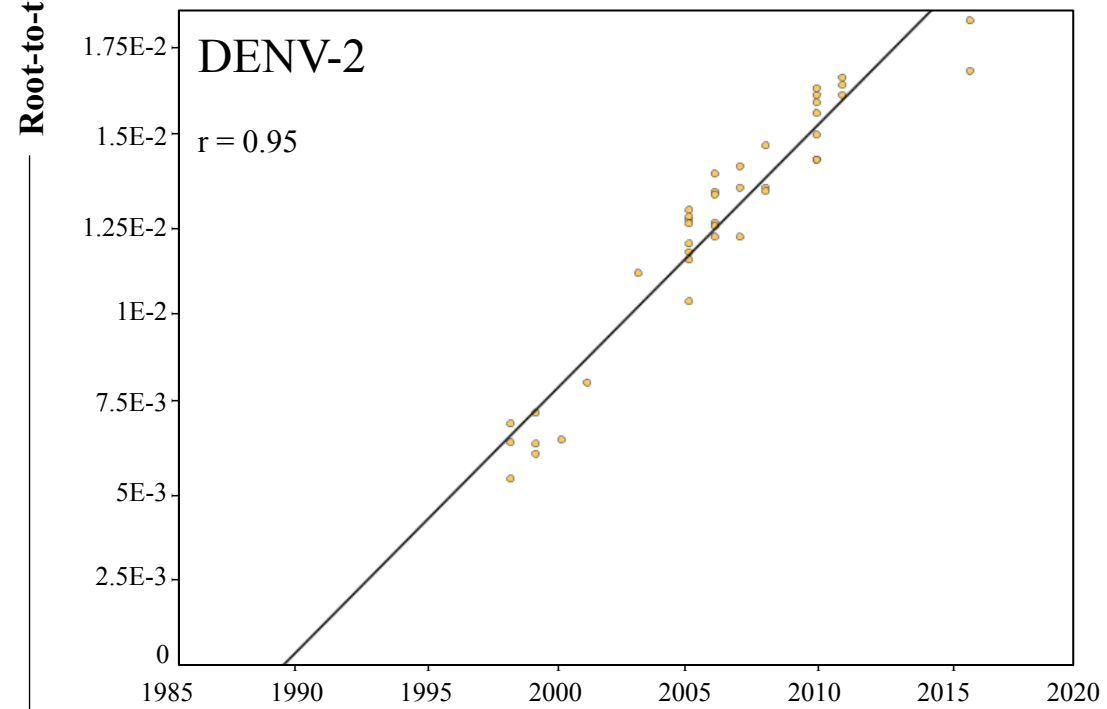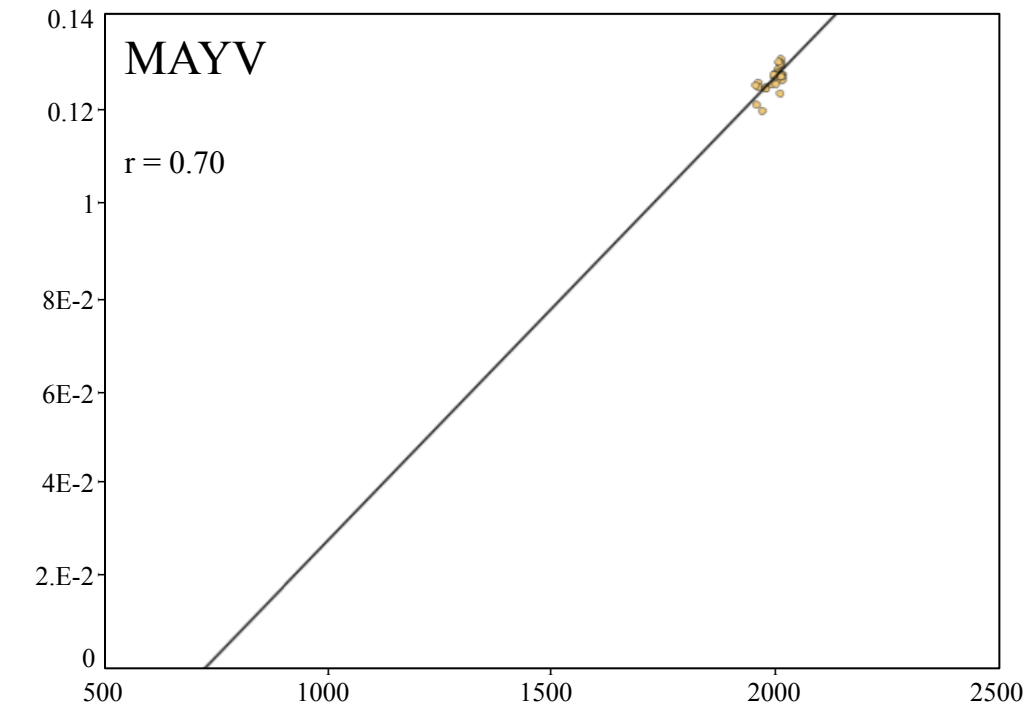

Supplement: S3 Fig — Midpoint rooted ML phylogenies for (A) CHIKV, (B) ZIKV, (C) DENV-2 and (D) MAYV. Branch lengths are scaled in nucleotide substitutions per site according to the bar at the bottom of each tree. Tips are colored by sampling location as indicated in the legends to the left of each tree; diamonds at each node indicate strong statistical support along branches defined as ultrafast bootstrapping>90% (out of 2,000 replicates). (PDF) [file pntd.0006505.s004.pdf]
