# Supplementary material for: Detection and phylogenetic characterization of arbovirus dual-infections among persons during a chikungunya fever outbreak, Haiti 2014
Source: PLoS Negl Trop Dis. 2018 May 31;12(5):e0006505. doi: 10.1371/journal.pntd.0006505 (PMC5997359; doi:10.1371/journal.pntd.0006505)
Supplement: S1 Table — (DOCX) [file pntd.0006505.s005.docx]

**SUPPLEMENTARY TABLES**

**Table S1.** ZIKV, DENV-2 and MAYV genomic sequence information.

| GenBank accession | Virus | Geographical location | Year |
| --- | --- | --- | --- |
| KX702404 | DENV-2 | USA,Florida | 2016 |
| KY415991 | DENV-2 | Haiti | 2016 |
| EU482546 | DENV-2 | Puerto Rico | 1998 |
| EU482560 | DENV-2 | Puerto Rico | 1998 |
| EU596491 | DENV-2 | Puerto Rico | 2007 |
| EU677142 | DENV-2 | Puerto Rico | 1999 |
| EU677145 | DENV-2 | Puerto Rico | 1999 |
| EU687212 | DENV-2 | Puerto Rico | 1998 |
| EU687216 | DENV-2 | Puerto Rico | 2005 |
| EU687217 | DENV-2 | Puerto Rico | 2005 |
| EU920832 | DENV-2 | French Guiana | 1999 |
| EU920833 | DENV-2 | French Guiana | 2000 |
| EU920836 | DENV-2 | Martinique | 2005 |
| EU920839 | DENV-2 | Martinique | 2005 |
| EU920841 | DENV-2 | Martinique | 2005 |
| EU920843 | DENV-2 | Martinique | 2005 |
| EU920842 | DENV-2 | Suriname | 2005 |
| EU920838 | DENV-2 | Suriname | 2005 |
| EU920844 | DENV-2 | French Guiana | 2006 |
| EU920845 | DENV-2 | French Guiana | 2006 |
| EU920846 | DENV-2 | French Guiana | 2006 |
| EU920847 | DENV-2 | French Guiana | 2006 |
| EU920849 | DENV-2 | Guadeloupe | 2006 |
| EU920850 | DENV-2 | Guadeloupe | 2006 |
| FJ898451 | DENV-2 | Dominican Republic | 2003 |
| FJ898453 | DENV-2 | Virgin Islands | 2005 |
| FJ898460 | DENV-2 | Saint Kitts and Nevis | 2001 |
| GQ199892 | DENV-2 | Jamaica | 2007 |
| FJ850091 | DENV-2 | Brazil | 2007 |
| GQ199890 | DENV-2 | Brazil | 2008 |
| GU131864 | DENV-2 | Brazil | 2008 |
| HQ026763 | DENV-2 | Brazil | 2008 |
| JX286516 | DENV-2 | Brazil | 2010 |
| JX286522 | DENV-2 | Brazil | 2010 |
| JX286526 | DENV-2 | Brazil | 2010 |
| JX669479 | DENV-2 | Brazil | 2010 |
| KC294202 | DENV-2 | Peru | 2010 |
| KC294203 | DENV-2 | Peru | 2010 |
| KC294204 | DENV-2 | Peru | 2011 |
| KC294206 | DENV-2 | Peru | 2011 |
| KC294207 | DENV-2 | Peru | 2011 |
| KC294223 | DENV-2 | Peru | 2010 |
| KX496990 | MAYV | Haiti | 2015 |
| KT754168 | MAYV | Pará, Brazil | 1960 |
| KT818520 | MAYV | São Paulo Brazil | 2014 |
| KP842820 | MAYV | Pará, Brazil | 1961 |
| KP842819 | MAYV | Pará, Brazil | 1955 |
| KP842818 | MAYV | Pará, Brazil | 1991 |
| KP842817 | MAYV | Bolivia | 2006 |
| KP842816 | MAYV | Iquitos, Peru | 2011 |
| KP842815 | MAYV | Iquitos, Peru | 2011 |
| KP842814 | MAYV | Bolivia | 2006 |
| KP842813 | MAYV | Yurimaguas, Peru | 2011 |
| KP842812 | MAYV | Puerto Maldonado, Peru | 2010 |
| KP842811 | MAYV | Puerto Maldonado, Peru | 2005 |
| KP842810 | MAYV | Trinidad and Tobago | 1957 |
| KP842809 | MAYV | Brazil | 1970 |
| KP842808 | MAYV | Loreto, Peru | 2000 |
| KP842807 | MAYV | Loreto, Peru | 1995 |
| KP842806 | MAYV | Bolivia | 2006 |
| KP842805 | MAYV | Bolivia | 2002 |
| KP842804 | MAYV | Pará, Brazil | 1978 |
| KP842803 | MAYV | Pará, Brazil | 1978 |
| KP842802 | MAYV | Pará, Brazil | 1978 |
| KP842801 | MAYV | Loreto, Peru | 2006 |
| KP842800 | MAYV | San Martin, Peru | 1995 |
| KP842799 | MAYV | La Estación Portuguesa, Venezuela | 2010 |
| KP842798 | MAYV | La Estación Portuguesa, Venezuela | 2010 |
| KP842797 | MAYV | La Estación Portuguesa, Venezuela | 2010 |
| KP842796 | MAYV | La Estación Portuguesa, Venezuela | 2010 |
| KP842795 | MAYV | La Estación Portuguesa, Venezuela | 2010 |
| KP842794 | MAYV | La Estación Portuguesa, Venezuela | 2010 |
| KM400591 | MAYV | Acre, Brazil | 2004 |
| KJ013266 | MAYV | French Guiana | 2013 |
| DQ001069 | MAYV | French Guiana | 1999 |
| KX447509 | ZIKV | French Polynesia | 2013 |
| KX447517 | ZIKV | French Polynesia | 2014 |
| KX369547 | ZIKV | French Polynesia | 2013 |
| KJ776791 | ZIKV | French Polynesia | 2013 |
| KY014302 | ZIKV | Dominican Republic | 2016 |
| KY014314 | ZIKV | Dominican Republic | 2016 |
| KY014304 | ZIKV | Dominican Republic | 2016 |
| KY325464 | ZIKV | USA, Florida | 2016 |
| KY325473 | ZIKV | USA, Florida | 2016 |
| KX838904 | ZIKV | USA, Florida | 2016 |
| KX922703 | ZIKV | USA, Florida | 2016 |
| KX922707 | ZIKV | USA, Florida | 2016 |
| FL001Sa | ZIKV | USA, Florida | 2016 |
| FL016U | ZIKV | USA, Florida | 2016 |
| FL008U | ZIKV | USA, Florida | 2016 |
| KY325465 | ZIKV | USA, Florida | 2016 |
| KX520666 | ZIKV | Brazil | 2015 |
| KU321639 | ZIKV | Brazil | 2015 |
| KX830930 | ZIKV | Brazil | 2016 |
| KU729218 | ZIKV | Brazil | 2015 |
| KU365778 | ZIKV | Brazil | 2015 |
| KU926309 | ZIKV | Brazil | 2016 |
| FC-6418-SER | ZIKV | Brazil | 2016 |
| FC-DQ62D2-URI | ZIKV | Brazil | 2016 |
| KX269878 | ZIKV | Haiti | 2016 |
| KX051563 | ZIKV | Haiti | 2016 |
| KU509998 | ZIKV | Haiti | 2014 |
| KY415986 | ZIKV | Haiti | 2014 |
| KY415987 | ZIKV | Haiti | 2014 |
| KY415988 | ZIKV | Haiti | 2014 |
| KY415989 | ZIKV | Haiti | 2014 |
| KY415990 | ZIKV | Haiti | 2014 |
| KY415991 | ZIKV | Haiti | 2014 |
| KX673530 | ZIKV | Guadeloupe | 2016 |
| KU758877 | ZIKV | French Guiana | 2015 |
| KU937936 | ZIKV | Suriname | 2015 |
| KU312312 | ZIKV | Suriname | 2015 |
| KY693676 | ZIKV | Honduras | 2016 |
| KY328289 | ZIKV | Honduras | 2016 |
| HU-ME152-SER | ZIKV | Honduras | 2016 |
| KX766029 | ZIKV | Mexico | 2016 |
| KY631493 | ZIKV | Mexico | 2015 |
| KU922923 | ZIKV | Mexico | 2016 |
| KY693678 | ZIKV | Peru | 2016 |
| KY693679 | ZIKV | Peru | 2016 |
| KX702400 | ZIKV | Venezuela | 2016 |
| KY693680 | ZIKV | Venezuela | 2016 |
| KX879603 | ZIKV | Ecuador | 2016 |
| KX879604 | ZIKV | Ecuador | 2016 |
| KX377337 | ZIKV | Puerto Rico | 2015 |
| KU501215 | ZIKV | Puerto Rico | 2015 |
| KX087101 | ZIKV | Puerto Rico | 2015 |
| KU647676 | ZIKV | Martinique | 2015 |
| KU870645 | ZIKV | Guatemala | 2016 |
| KU501217 | ZIKV | Guatemala | 2015 |
| KU501216 | ZIKV | Guatemala | 2015 |
| KX548902 | ZIKV | Colombia | 2015 |
| KU820897 | ZIKV | Colombia | 2015 |
| KX247646 | ZIKV | Colombia | 2016 |
| KX156775 | ZIKV | Panama | 2015 |
| KX156774 | ZIKV | Panama | 2015 |
| KX198135 | ZIKV | Panama | 2016 |
| AB860301 | CHIKV | Philippines | 2013 |
| KJ451624 | CHIKV | British Virgin Islands | 2014 |
| KP164567 | CHIKV | Brazil | 2014 |
| KP164571 | CHIKV | Brazil | 2014 |
| KP164572 | CHIKV | Brazil | 2014 |
| KP851709 | CHIKV | Mexico | 2014 |
| KP851710 | CHIKV | Mexico | 2014 |
| KR046227 | CHIKV | Trinidad and Tobago | 2014 |
| KR046228 | CHIKV | Trinidad and Tobago | 2014 |
| KR046229 | CHIKV | Trinidad and Tobago | 2014 |
| KR046230 | CHIKV | Trinidad and Tobago | 2014 |
| KR046231 | CHIKV | Trinidad and Tobago | 2014 |
| KR046232 | CHIKV | Trinidad and Tobago | 2014 |
| KR046233 | CHIKV | Trinidad and Tobago | 2014 |
| KR046234 | CHIKV | Trinidad and Tobago | 2014 |
| KR264949 | CHIKV | Puerto Rico | 2014 |
| KR264951 | CHIKV | Puerto Rico | 2014 |
| KR559470 | CHIKV | Puerto Rico | 2014 |
| KR559471 | CHIKV | El Salvador | 2014 |
| KR559472 | CHIKV | El Salvador | 2014 |
| KR559473 | CHIKV | French Polynesia | 2015 |
| KR559474 | CHIKV | Puerto Rico | 2014 |
| KR559475 | CHIKV | El Salvador | 2014 |
| KR559476 | CHIKV | Haiti | 2014 |
| KR559477 | CHIKV | Dominican Republic | 2014 |
| KR559478 | CHIKV | Haiti | 2014 |
| KR559479 | CHIKV | Dominican Republic | 2014 |
| KR559480 | CHIKV | US Virgin Islands | 2014 |
| KR559481 | CHIKV | Guatemala | 2014 |
| KR559482 | CHIKV | US Virgin Islands | 2014 |
| KR559483 | CHIKV | Puerto Rico | 2014 |
| KR559484 | CHIKV | El Salvador | 2014 |
| KR559485 | CHIKV | US Virgin Islands | 2014 |
| KR559486 | CHIKV | Panama | 2014 |
| KR559487 | CHIKV | Honduras | 2014 |
| KR559488 | CHIKV | Honduras | 2014 |
| KR559489 | CHIKV | Jamaica | 2014 |
| KR559490 | CHIKV | Guyana | 2014 |
| KR559491 | CHIKV | Colombia | 2014 |
| KR559492 | CHIKV | Saint Lucia | 2014 |
| KR559494 | CHIKV | US Virgin Islands | 2014 |
| KR559495 | CHIKV | Puerto Rico | 2014 |
| KR559496 | CHIKV | Guyana | 2014 |
| KR559498 | CHIKV | Dominican Republic | 2014 |
| KT192707 | CHIKV | Nicaragua | 2014 |
| KT308159 | CHIKV | Philippines | 2012 |
| KT308160 | CHIKV | Philippines | 2012 |
| KT308162 | CHIKV | Philippines | 2012 |
| KT327163 | CHIKV | Mexico | 2014 |
| KT327164 | CHIKV | Mexico | 2014 |
| KT327165 | CHIKV | Mexico | 2014 |
| KT327166 | CHIKV | Mexico | 2014 |
| KT327167 | CHIKV | Mexico | 2014 |
| KX262991 | CHIKV | Saint Martin | 2003 |
| KX262992 | CHIKV | Guadeloupe | 2014 |
| KX262994 | CHIKV | French Guinea | 2014 |
| KX496989 | CHIKV | Colombia | 2016 |
| KX702401 | CHIKV | Haiti | 2014 |
| KX702402 | CHIKV | Haiti | 2014 |
| KY415978 | CHIKV | Haiti | 2014 |
| KY415979 | CHIKV | Haiti | 2014 |
| KY415980 | CHIKV | Haiti | 2014 |
| KY415981 | CHIKV | Haiti | 2014 |
| KY415982 | CHIKV | Haiti | 2014 |
| KY415983 | CHIKV | Haiti | 2014 |
| KY415984 | CHIKV | Haiti | 2014 |
| KY415985 | CHIKV | Haiti | 2014 |
| LN898093 | CHIKV | Martinique | 2013 |
