# Supplementary material for: Detection and phylogenetic characterization of arbovirus dual-infections among persons during a chikungunya fever outbreak, Haiti 2014
Source: PLoS Negl Trop Dis. 2018 May 31;12(5):e0006505. doi: 10.1371/journal.pntd.0006505 (PMC5997359; doi:10.1371/journal.pntd.0006505)
Supplement: S2 Table — (DOCX) [file pntd.0006505.s006.docx]

Table S2. Bayes factor (BF) comparison of nested molecular clock and Bayesian demographic models.

| **CHIKV** | SC Constant | UCLN Constant | SC  BSP | UCLN BSP |  |  |  |  |  |
| --- | --- | --- | --- | --- | --- | --- | --- | --- | --- |
| SC Constant |  | 1.960 | 15.437 | - | Path sampling |  |  |  |  |
| UCLN Constant | 1.879 |  | - | 16.474 |  |  |  |  |  |
| SC  BSP | 15.397 | - |  | 2.997 |  |  |  |  |  |
| UCLN BSP | - | 16.446 | 2.928 |  |  |  |  |  |  |
|  | Stepping stone sampling | | | |  |  |  |  |  |
| **ZIKV** | SC Constant | UCLN Constant | SC  BSP | UCLN BSP |  |  |  |  |  |
| SC Constant |  | -0.058 | 24.137 | - | Path sampling |  |  |  |  |
| UCLN Constant | -0.105 |  | - | 26.946 |  |  |  |  |  |
| SC  BSP | 23.823 | - |  | 2.751 |  |  |  |  |  |
| UCLN BSP | - | 26.916 | 2.987 |  |  |  |  |  |  |
|  | Stepping stone sampling | | | |  |  |  |  |  |
| **DENV-2** | SC Constant | UCLN Constant | SC  BSP | UCLN BSP |  |  |  |  |  |
| SC Constant |  | 10.868 | 0.084 | - | Path sampling |  |  |  |  |
| UCLN Constant | 10.819 |  | - | -1.983 |  |  |  |  |  |
| SC  BSP | 0.008 | - |  | 8.800 |  |  |  |  |  |
| UCLN BSP | - | -2.049 | 8.760 |  |  |  |  |  |  |
|  | Stepping stone sampling | | | |  |  |  |  |  |
| **MAYV** | SC Constant | UCLN Constant | SC  BSP | UCLN BSP |  |  |  |  |  |
| SC Constant |  | 14.231 | 0.752 | - | Path sampling |  |  |  |  |
| UCLN Constant | 14.069 |  | - | 4.955 |  |  |  |  |  |
| SC  BSP | 0.396 | - |  | 18.433 |  |  |  |  |  |
| UCLN BSP | - | 5.522 | 19.225 |  |  |  |  |  |  |
|  | Stepping stone sampling | | | |  |  |  |  |  |
